# Syndemic Factors Associated with Zika Virus Infection Prevalence and Risk Factors in a Cohort of Women Living in Endemic Areas for Arboviruses in Northeast Brazil

**DOI:** 10.3390/tropicalmed10030067

**Published:** 2025-03-01

**Authors:** Ligia Kerr, Carlos Sanhueza-Sanzana, Marto Leal, Italo Aguiar, Kasim Allel, Moisés H. Sandoval, Cristiane Cunha Frota, Marco Túlio Aguiar, Adriano Ferreira Martins, Livia Dias, Rosa Livia Freitas de Almeida, Francisco Herlânio Costa Carvalho, Francisco Gustavo Silveira Correia, Roberto da Justa Pires Neto, Fernanda Montenegro Araújo, Shirlene Telmos Silva de Lima, Leda Maria Simões Mello, Lucas de Lima Nogueira, Terezinha do Menino Jesus Silva Leitão, Maria da Glória Teixeira, Jeni Stolow, Guilherme Loureiro Werneck, Ivo Castelo Branco Coelho, Ronald Blanton, Ana Zaira da Silva, George W. Rutherford, Carl Kendall

**Affiliations:** 1 Departamento de Saúde Comunitária, Universidade Federal do Ceará, Av. Prof. Costa Mendes 1608, Edifício Didático, 5° Andar, Rodolfo Teófilo, Fortaleza 60430-160, CE, Brazil; carlosanhueza.san@gmail.com (C.S.-S.); marcotuliomfc@gmail.com (M.T.A.); adrianoenfobr@gmail.com (A.F.M.); liviadiasenf@gmail.com (L.D.); rliviafa@gmail.com (R.L.F.d.A.); herlaniocosta@gmail.com (F.H.C.C.); gustavcorreia@gmail.com (F.G.S.C.); robertojusta@gmail.com (R.d.J.P.N.); tsilva@ufc.br (T.d.M.J.S.L.); ivocastelo@uol.com.br (I.C.B.C.); ana.zaira@univasf.edu.br (A.Z.d.S.); carl.kendall@gmail.com (C.K.); 2 Nuffield Department of Population Health Sciences, University of Oxford, Woodstock Road, Oxford OX2 6GG, UK; kasim.allelhenriquez@phc.ox.ac.uk; 3 Department of Disease Control, London School of Hygiene and Tropical Medicine, Keppel St., London WC1E 7HT, UK; 4 Instituto de Nutrición y Tecnología de Alimentos Doctor Fernando Monckeberg Barros, Universidad de Chile, El Líbano 5524, Macul 7810000, Chile; msandoval@inta.uchile.cl; 5 Departamento de Patologia e Medicina Legal, Universidade Federal do Ceará, Av. Prof. Costa Mendes 1608, Edifício Didático, 5° Andar, Rodolfo Teófilo, Fortaleza 60430-160, CE, Brazil; cristianefrota71@gmail.com (C.C.F.); lucas.nogueira@ufc.br (L.d.L.N.); 6 Laboratório de Saúde Pública do Estado do Ceará (LACEN), Av. Barão de Studart, Fortaleza 60120-002, CE, Brazil; fernandamontenegrocaraujo@gmail.com (F.M.A.); shtlima73@gmail.com (S.T.S.d.L.); ledamsimoes@gmail.com (L.M.S.M.); 7 Instituto de Saúde Coletiva, Universidade Federal da Bahia, Rua Brasílio da Gama, s/n, Salvador 40110-040, BA, Brazil; t.gloria@hotmail.com; 8 Department of Social Behavioral and Population Sciences, Tulane Celia Scott Weatherhead School of Public Health and Tropical Medicine, 1440 Canal Street, New Orleans, LA 70112, USA; jeni.stolow@gmail.com; 9 Instituto de Estudos em Saúde Coletiva, Universidade Federal do Rio de Janeiro, Av. Horácio Macedo, s/n, Cidade Universitária, Rio de Janeiro 21941-598, RJ, Brazil; gwerneck@iesc.ufrj.br; 10 Instituto de Medicina Social, Universidade do Estado do Rio de Janeiro, Rua São Francisco Xavier 524, Maracanã, Bloco E, 7°eandar, Rio de Janeiro 20550-013, RJ, Brazil; 11 Department of Tropical Medicine, School of Public Health and Tropical Medicine, Tulane University, 1440 Canal Street, New Orleans, LA 70112, USA; rblanton1@tulane.edu; 12 Institute for Global Health Sciences, University of California, 550 16th Street, San Francisco, CA 94158, USA; george.rutherford@ucsf.edu

**Keywords:** Zika virus infection, arboviruses, syndemic, women’s health, prevalence, Brazil

## Abstract

Background: We sought to explain the seroprevalence of Zika Virus (ZIKV) as a syndemic of socioeconomic, environmental, and health factors in a cohort of women living in Brazil. Methods: This is a cohort study comprising 1498 women between 15 and 39 years of age followed up in two waves between February 2018 and August 2019. Two questionnaires addressed the arbovirus’s socioeconomic, demographic, and behavioral aspects and participants’ arbovirus infection history. Blood samples were collected to detect IgM and IgG for ZIKV, chikungunya virus (CHIKV), and dengue virus (DENV), and RT-PCR for ZIKV. Results: The baseline prevalence for ZIKV was 43% (95%CI: 40.5, 45.6), increasing to 44.7% in the following period (95%CI: 42, 47.1). We found a prevalence of 44.1% among women having one syndemic factor, 49.9% for those having two, and 58% for women having three or more factors. Women reporting a single syndemic factor resulted in higher odds of acquiring ZIKV (OR = 1.6, 95%CI: 1.2–2.4). There were increased adjusted odds among women having two or three or more factors (OR = 2.1, 95%CI: 1.6–3.1; OR = 2.9, 95%CI: 2.0–4.3, respectively). Conclusions: Tailored interventions targeting syndemic conditions, such as the co-circulation of urban arboviruses and poor living conditions, are crucial to improving the burden produced by ZIKV.

## 1. Introduction

Vector-borne diseases, like arboviruses, continue to be a major contributor to the global burden of disease [1]. The increasing distribution of *Aedes aegypti,* the main vector of arboviruses like dengue, chikungunya, yellow fever, and Zika, has been driven by the displacement of human populations and rising temperatures due to climate change [2]. Climate change expands the range and accelerates the life cycle of *Aedes aegypti*, promoting the spread of the vector and the disease in previously unaffected areas [2,3]. As such, they represent a serious threat to the lives of almost half of the world’s population [4].

In 2022, 3,125,367 cases of arboviruses were reported in the Americas: 90% of cases were dengue cases, 8.7% were chikungunya, and 1.3% were Zika cases. Those more than 3 million cases of arboviruses represent an increase of almost 120% compared to the same period in 2021 (~1.5 million cases) [5].

Zika virus emerged as a global public health concern in 2015 and continues to pose significant risks to women of childbearing age worldwide. The virus gained widespread attention due to its association with severe birth defects, notably microcephaly, in infants born to infected mothers [6]. The impact of Zika on women of reproductive age extends beyond the risk of congenital malformations, raising crucial questions about reproductive health, family planning, and overall well-being [7].

The Zika epidemic in Brazil, which began in 2015, generated global concern due to the high number of reported cases and the subsequent rise in microcephaly [8]. Brazil’s tropical climate, densely populated and impoverished urban areas, and abundant *Aedes* mosquito populations provided favorable conditions for Zika transmission [9,10]. In 2022, 9204 probable cases of Zika were registered in the country, with the Northeast Region, which is one of the poorest regions in the country, having the highest incidence (13.3 cases/100,000 inhabitants) [11]. Furthermore, northeast Brazil reported the most cases of congenital Zika syndrome (CZS) [11]. The association between ZIKV infection and poverty, such as poor housing, inadequate water supplies, lower educational level, poor sanitation and hygiene [12,13], inadequate food consumption [14], and previous coinfection with other arboviral infections [15], provide evidence of the impact of social inequalities on health [16].

In northeast Brazil, the Zika epidemic triggered a public health system crisis, placing immense strain on healthcare systems and challenging existing infrastructure. Women of reproductive age were particularly vulnerable, as they found themselves at the intersection of Zika’s potential consequences for maternal and child health and the constraints on women’s reproductive autonomy and health system support, either for abortion or ameliorative child development interventions for affected children [17,18].

Although there has been a significant reduction in the number of ZIKV infections in recent years, Zika, chikungunya, and dengue have occurred in epidemic waves in several areas of the country, either together or separately. This co-infection and the intersection of poverty, gender, and social norms lends itself to the examination of the problem as a syndemic: multiple epidemics of disease, economic and social conditions, and physical environment, each multiplying the individual effects of these factors. Singer characterizes syndemics: “A syndemic … involves a set of enmeshed and mutually enhancing health problems that, working together in a context of noxious social and physical conditions, can significantly affect the overall disease burden and health status of a population” [19]. Observing the epidemic from this perspective argues for a comprehensive approach in response: one that takes into account not just parental support but broader efforts at vector control, housing, water management and sanitation, and especially women’s reproductive health and freedom.

The objective of this study was to explore these factors in a cohort of women of childbearing age from an area periodically affected by dengue, chikungunya, and Zika in Fortaleza, Ceará, the capital city of one of the states in the northeast region that has been deeply affected by cases of Zika and congenital Zika syndrome (CZS).

## 2. Materials and Methods

### 2.1. Study Population

Our study draws on a larger cohort, “Zika in Fortaleza: Response of a cohort of women aged 15–39 (ZIF)”. We recruited women of reproductive age (15–39 years) who lived in areas highly vulnerable to arbovirus infection in Fortaleza, Ceará, Brazil, attending one of four selected primary health care units (PHCUs).

Fortaleza is the largest city and capital of the state of Ceará. In 2017, the state had the highest incidence rate of chikungunya (1271.0/100,000 inhabitants), the second highest incidence rate of dengue (452.9/100 thousand inhabitants), and the fifth highest incidence rate of Zika (16.8/100,000 inhabitants) in the country [20]. The city has one of the highest population densities in Brazil (7786.44 inhabitants/km^2^). With an estimated population of 2,669,342 people [21], the city has 96 PHCU, among which 12 were staffed by physicians from the Federal University of Ceará’s Family and Community Health’s Medical Residency Program. Four of these PHCUs were chosen to participate in ZIF using geographic areas with the highest reported number of CHIKV cases reported in 2017 as a proxy for Zika virus distribution [22].

Two of these PHCU are located in the Barra do Ceará, an urban neighborhood located in the second most densely populated area of Fortaleza (Figure S1). The area has a very low human development index (HDI = 0.22), with poor infrastructure, lack of sanitation, difficulty in accessing water, unpaved streets, and solid household waste, all of which are favorable conditions for the proliferation of *Aedes aegypti*. The third PHCU is located in the Esperança neighborhood, which has a population of 16,405 inhabitants and an HDI of 0.29. The fourth PHCU is located in the area of Rodolfo Teófilo, which has 19,114 inhabitants and an HDI of 0.48, the highest of the four units.

Inclusion criteria for the participants were women who were (1) living in one of the chosen PHCU catchment areas; (2) aged 15–39 years (because of the higher probability of having a pregnancy); (3) sexually active (at least one sexual relationship during the last 12 months); (4) no tubal ligation or health problem that would affect pregnancy; and (5) agreeing to participate in the study. The sample size was calculated based on the following parameters: the probability of becoming pregnant estimated at 8.3% (±2) (total live births divided by the estimated sexually active female population multiplied by 100); a design effect (d_eff_) of 2; a 95% confidence interval (CI) calculated using the formula Z^2^_1_ − α/2 and 20% for loss to follow-up. The final sample size was 1752 women. During the data collection, we decided to reduce the sample size since the pregnancy rate (20%) for the women in our sample was much higher than we expected.

### 2.2. Data Collection

This study consisted of two cohort waves. The first was collected from 28 February to 30 October 2018, and the second was conducted from November 2018 to August 2019. In the second wave, we interviewed a total of 1176 women, a loss of 21.5% compared to the baseline.

**Figure 1:**
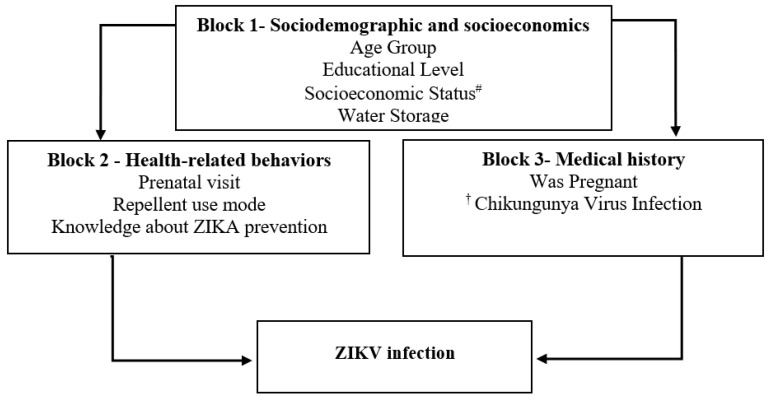
Theoretical framework of a hierarchical explanatory model for ZIKV infection in a cohort of women of reproductive age, Fortaleza, Brazil, 2018–2019. Notes: ^#^ ABEP Brazilian Association of Research Organizations. ^†^ Self-report of family history of arbovirus infection chikungunya virus, dengue virus, or Zika virus.

A semi-structured questionnaire was used to collect the data from the participants using the following explanatory blocks: Block 1—Socioeconomic and demographic factors (age; educational level; socioeconomic status; water storage); Block 2—Health-related behaviors (prenatal visit; repellent use mode; knowledge about Zika prevention); Block 3—Medical history (was pregnant; Chikungunya virus infection) (see Figure 1). A computer-assisted personal interview was performed with Survey Monkey software (Survey Monkey, Inc., Palo Alto, CA, USA) to collect information for the questionnaire.

### 2.3. Laboratory Tests

We collected two 5 mL vacutainer tubes (without anticoagulant) of peripheral venous blood from each participant. Blood specimens were collected regardless of pregnancy status or if the participant had symptoms of fever, rash, or other signs of arbovirus infection. Sera were separated by centrifugation at 1000× *g* for 10 min, aliquoted, and stored at −20 °C and −70 °C for ELISA. Specimens were tested for IgM and IgG anti-ZIKV, DENV and CHIKV at the Ceará State Reference Public Health Laboratory (LACEN-CE) and Mycobacterium Laboratory (School of Medicine, Federal University of Ceará) using EUROIMMUN ELISA kit (EUROIMMUN Medizinische Labordiagnostika AG, Lübeck, Germany), according to the manufacturer’s instructions and previous publications [23]. The reaction was stopped by adding 100 µL/well 0.5 M H_2_SO_4_ and waiting 30 min. Microplates were read at 450 nm on an ELISA reader (Wuxi Hiwell Diatek Instruments, Wuxi, China). Euroimmun IgM/IgG assays are widely used commercial ELISA tests. They have demonstrated high sensitivity, from 92.3% to 100% sensitivity, allowing earlier detection [24,25]. Cross-reactivities with other flaviviruses were overcome using differential diagnosis and algorithm interpretation. Indeterminate results were repeated.

**Figure 2:**
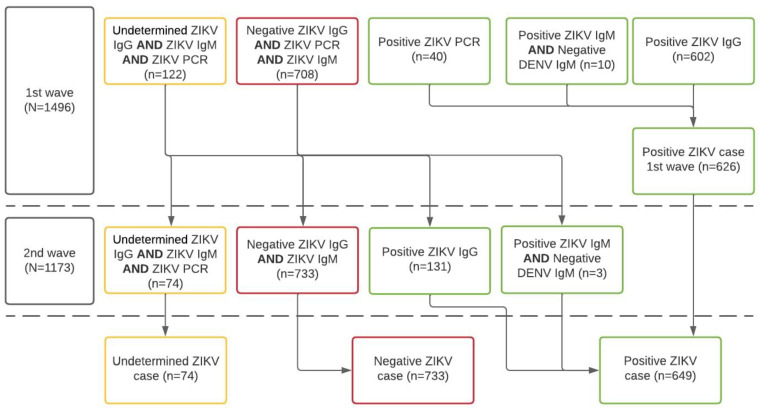
Algorithm for case definition of ZIKV infection according to laboratory results of the ZIF-cohort, Fortaleza, Brazil, 2018–2019. Notes: IgM or IgG anti-ZIKV, and anti-DENV, or RT-PCR anti-ZIKV and anti-DENV; N.B. 2nd wave tests produced fewer indeterminate results and women were reclassified according to the new lab results following the algorithm.

Using an enzyme-linked immunosorbent assay (ELISA), a recent case of ZIKV infection was defined by all those women having samples of ZIKV-specific IgM antibodies detected in serum samples. Previous ZIKV infections were defined as women having ZIKV-specific IgG antibodies. CHIKV infection was defined using the same standard definition (i.e., IgM and IgG). As sensitivity analyses, we compared the differential diagnosis, as per tandem determination of IgM ELISA for both dengue and Zika Viruses, according to the C-algorithm of the Guidelines for Surveillance of Zika Virus (Figure 2) [26].

We needed to account for differences in diagnoses because participants were exposed to the simultaneous co-circulation of other arboviruses, which might enable cross-reactions with historic genetically related flavivirus infections (especially DENV).

Due to funding constraints, we only conducted RT-qPCR for ZIKV in the first wave. Total viral RNA was extracted from 140 µL of serum with the QIAamp Viral RNA Mini Kit (Qiagen NV, Hilden, Germany) according to the manufacturer’s recommendations. Extracted RNA was aliquoted and stored at −70 °C. The quality of each RNA sample was assessed by the 260/280 nm ratio. A 5 µL aliquot of RNA from each sample was used for the detection of viral RNA with the ZDC kit (Zika, dengue, and chikungunya) Multiplex RT-qPCR Assay (Bio-Rad Laboratories, Hercules, CA, USA). The ZDC kit consists of a single multiplex reaction for the three viruses, performed according to the manufacturer’s recommendations and previous publications [27,28]. Assays were performed on the Applied Biosystems 7500 Fast Real-Time PCR System (Applied Biosystems, Waltham, MA, USA) using the fluorophores ZIKV-FAM, CHIKV-HEX, DENV-Texas Red, and Internal Control-Cy5. The 25 μL reaction mix consisted of iTaqTM Universal Probes One-Step Reaction Mix, iScriptTM Reverse Transcriptase, ZDC Multiplex PCR Assay Mix, viral nucleic acid template, and water. The amplification conditions were as follows: step 1 at 50 °C for 15 min; step 2 at 94 °C for 2 min; and step 3 of 45 cycles of 94 °C for 15 s, 55 °C for 40 s, and 68 °C for 30 s. In each run, negative (no template) and positive controls for ZIKV, DENV, and CHIKV were added. In addition, internal positive controls for ZIKV, DENV, and CHIKV were added to each sample. We interpreted the results as positive if the reaction generated an exponential curve that crossed the threshold set manually at each run. The ZIKV result was considered positive when the reaction generated an exponential curve that crossed the defined threshold at ≤38.50 cycles, while DENV was defined as positive if the crossing threshold (Ct) ≤ 37.36 [27].

Our study protocol was approved by the Committee of Ethics of the Federal University of Ceará (#2,497,069). All women who were eligible and participated in the study read and signed the Free and Informed Consent Form (ICF) before the collection of clinical data and biological samples.

### 2.4. Statistical Analysis

Our analytical sample consisted of 1382 women after eliminating indeterminate laboratory results and missing information for ZIKV, CHIKV, and DENV (missing data = 7.6%). We calculated the prevalence of ZIKV in both waves. The descriptive analysis compared the absolute and relative frequencies of the variables associated with the prevalence of ZIKV. We used Pearson’s Chi-square test, utilizing a significance level of *p* < 0.05.

We adapted a previously proposed conceptual framework to analyze our data [29,30] (Figure 1). Bivariate analyses were performed for all variables in the three blocks. Block Number 1 was the first to be analyzed; as socioeconomic and demographic variables, they are considered the most distal in the transmission of Zika. The variables that were statistically significant at the 0.05 level in the final multivariate analysis for this block remained in the final model. For the other blocks, the variables that remained significant up to 0.20 were examined in a multivariate analysis internal to the block. In the final model, all variables that were statistically significant, adjusted for socioeconomic and demographic variables, were considered determinants of Zika infection.

The last step of the analysis was to identify syndemic factors associated with an increased probability of ZIKV infection, using univariate and multivariate logistic regression models. The term syndemic is used to describe the cumulative effect of one or more diseases that occur in the same time period and space, commonly within socioenvironmental contexts of inequality that promote the grouping of conditions and greater vulnerabilities in disadvantaged populations, according to Singer’s definition [15]. Following analysis, we considered the syndemic factors in our analysis as follows: (1) having incomplete elementary education or being illiterate, (2) storing water at home, and (3) having a family history of arbovirus infection (see below). The prevalence of neither, one, two, or three conditions co-occurring simultaneously were considered as syndemic factors for ZIKV infection.

We estimated predictive margins with their respective 95%CIs. We considered the results to be statistically significant at *p* < 0.05. All analyzes were performed using Stata Statistical Software: Release 16. (StataCorp LLC, College Station, TX, USA).

## 3. Results

### 3.1. Prevalence of ZIKV, DENV, and CHIKV Infection

The overall prevalence of past and current ZIKV infection in the first wave was 43% (95%CI: 40.5–45.6), while the prevalence in the second wave increased to 44.7% (95%CI 42–47.1) (Table 1). For CHIKV, we estimated prevalence to be 36.8% (95%CI: 34.3–39.2) in the first wave and 38.1% (95%CI: 35.6–40.6) in the second. DENV had the highest prevalence: 87% (95%CI: 85.1–89), and 88.7% (95%CI: 97–90.2) in the first and second wave, respectively (Table 1).

### 3.2. Factors Associated with ZIKV

Women aged between 20 and 29 or 30 and 39 years had 1.53 and 1.59 higher odds of ZIKV infection, respectively, compared with those aged 15 to 19 years (Table 2). Women with lower levels of education, who lived in disadvantaged socioeconomic backgrounds, had one or more children, had to store water in the house, had prior CHKV infections, had a self-reported family history of arbovirus infections, and reported incorrectly or did not use repellant had higher odds of ZIKV infection (Table 2). On the other hand, women who were pregnant at the time of the interview, visited prenatal care services or knew one or more ZIKV prevention had lower chance of ZIKV infection (Table 2).

The multivariate regression model showed that older age, lower educational attainment, water storage, lack of repellent usage, and previous history of CHIKV infection were found to be strong predictors of ZIKV infection. Pregnant women who reported prenatal visits to health care units were found to have lower odds of ZIKV infection (Table 3).

### 3.3. Syndemic Factors for ZIKV Infection

**Figure 3:**
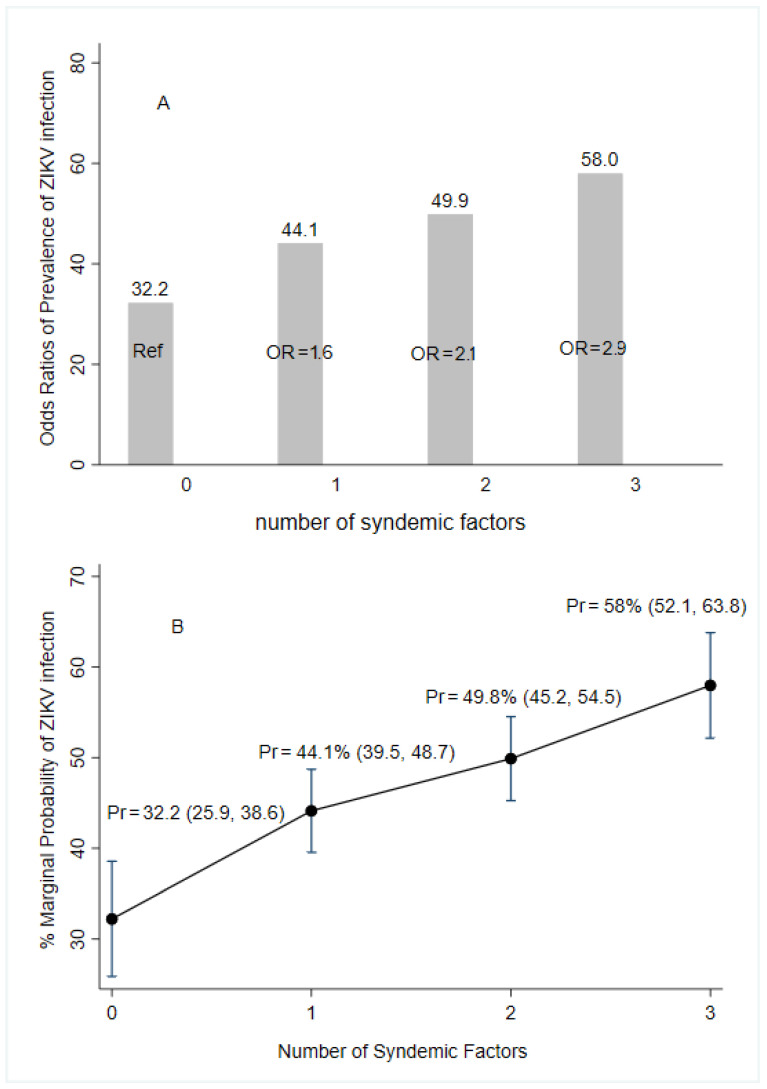
Predictive margins and marginal effect for syndemic factors of the prevalence of ZIKV infection according to laboratory results (following the algorithm for case definition in Figure 2), ZIF cohort, Fortaleza, Brazil, 2018–2019. Notes: OR (95%CI) = Odds Ratio (elementary complete or less education, water storage, and self-report of family history of arbovirus infections). Pr = marginal probability of multivariate logistic regression; Ref = reference category.

Worse environmental conditions such as water storage at home, self-reported family history of arboviruses, and lower educational attainment were associated with increased odds of acquiring ZIKV infections. The presence of only one factor had 1.6 times greater odds of infection while presenting two and three of the conditions had 2.1- and 2.9-times higher odds of infection, respectively, compared to those women presenting no syndemic factors (Figure 3).

## 4. Discussion

We found a prevalence of past or current ZIKV infection greater than 40% among our cohort of reproductive-age women in Ceará, Brazil. A systematic review that estimated the prevalence of asymptomatic Zika virus infection in the general population ranged from 23% in French Guiana to 60% in Zambia [31]. Our result is more than double the pooled prevalence of ZIKV infection across the world (18%; 95%CI: 12–25) but is consistent with prevalence observed in the region of the Americas (34%, 95%CI: 24–45), the highest reported among the WHO regions. Brazil was particularly affected and reported the highest prevalence of congenital Zika syndrome during this period [32]. Studies conducted among cohorts of pregnant women in Brazil reported a ZIKV prevalence of 61% in Recife [26] and Salvador [9] and 53% in Rio de Janeiro [33]. It is possible that the higher prevalence in these two cities is due to the population selected since pregnant women are more likely to be in prenatal care and more likely to be tested for arboviral infections.

To the best of our knowledge, this is the first study to show the syndemic association with co-circulation of arboviruses in a specific population of women of reproductive age living in areas vulnerable to ZIKV infection. We demonstrate how impoverished living conditions, low levels of education, and family history of previous arbovirus infection were associated with a higher prevalence of ZIKV. Our evidence highlights the high vulnerability of our targeted population due to low education and the poor living conditions of the women in our cohort.

Among the factors we found associated with a higher prevalence of ZIKV infection were poor housing conditions and water storage together with poverty and inadequate health and domestic hygiene. In contrast, women in our sample who were pregnant at the time of the interview and those in prenatal care who used repellent correctly and knew at least one ZIKV prevention method had lower adjusted odds of ZIKV infection. This is of special interest given that so much of health coverage and promotion, especially on TV, focused on microcephaly and the risks to pregnant women. Although not formally evaluated, this may have generated increased repellant use and other mosquito avoidance behaviors, generating our results.

Syndemic factors such as poor environmental conditions, low educational levels, and co-circulation of arboviruses have been shown to be strong predictors of poorer health levels in women of reproductive age. A syndemic approach recognizes that diseases in a population occur neither independently of social and ecological conditions nor in isolation from other diseases. There is constant interaction that can result in the clustering of diseases and enhancement at the community or population levels and direct biological interaction of disease pathologies at the individual and even cellular levels [15]. The growing literature on syndemics parallels the growing awareness of structural, social, and economic determinants of health. Certainly, ecological and human factors appear to play a role in determining the increased incidence of vector-borne diseases [34].

The Brazilian Surveillance System recommends conducting monitoring for the timely detection of DENV, CHIKV, and ZKV to detect possible changes in the pattern of circulation of these arboviruses [35]. Furthermore, the surveillance system guidelines also recommend monitoring the occurrence of Zika in pregnant women and cases of neurological manifestations possibly related to previous infection with these arboviruses. Like dengue and chikungunya, Zika should receive priority care in Primary Health Care. The Brazilian Surveillance System also recommends the installation of mosquito nets and protective structures at home, such as screens on windows and doors, and, especially for pregnant women, the use of clothes that minimize exposure and provide protection from vector bites, such as pants and long-sleeved shirts. These measures may be impractical due to the high temperature characteristic of these areas closer to the equator, which are also the most affected by arboviruses [18].

Future research, beyond monitoring the long-term effects of arbovirus infection on women’s health, should attempt to understand the mechanism of syndemic effects or at least translate these intersectional effects into intervention design. Simple recommendations for mosquito avoidance are not sufficient to interrupt the regular outbreaks of arbovirus infection in Brazil and elsewhere in the world where mosquitoes thrive. The best control efforts adopt a socio-ecological model [36] that integrates individual, community, city, and society-level interventions, focused on mosquito control, fever monitoring, health infrastructure, and enhanced care-seeking, household and community development with food security, and substantial societal engagement.

Our study has some shortcomings. RT-PCR for confirmation of ZIKV, DENV, and CHIKV were difficult to perform in the second wave due to cost restrictions that may lead to underestimation of arbovirus infections in our study’s second wave.

The strength of the paper is to show that the high prevalence of ZIKV in women living in vulnerable areas continues to be a public health problem in Brazil. ZIKV among women is mainly determined by syndemic conditions such as co-circulation of urban arboviruses and poor living conditions. Although the Ministry of Health has launched guidelines with recommendations for Zika-related care in the context of family planning, including prenatal and child care [37], there are still great obstacles faced by the most vulnerable women in obtaining information and access to effective contraceptive methods, including safe abortion [38]. Simultaneously, these recommendations are just launched as declarative messages rather than as part of systematic health education and promotion, missing opportunities to understand the syndemy of structural causes, community issues including failing infrastructure and services, the severity and causes of ongoing mosquito infestation, and other factors that might lead to effective change, individual and societal. Understanding the unique challenges faced by this population is essential for implementing effective strategies to mitigate the long-term implications of Zika.

## Figures and Tables

**Table 1:** Characteristics of women in the first and second wave of the ZIF cohort, Fortaleza, Brazil, 2018–2019.

Laboratory Variables	Wave 1 ^†^ (n = 1496)		Wave 2 ^†^ (n = 1173)	
	n (%)	95%CI	n (%)	95%CI
ZIKV infection				
IgG	602 (41.4)	(38.8–43.9)	131 (15.1)	(43.9–49.6)
IgM	10 (0.7)	(0.4–1.3)	5 (0.7)	(0.3–1.6)
RT-PCR	40 (3.1)	(2.3–4.2)	-	-
Total Prevalence ^††^	626 (43.0)	(40.5–45.6)	649 (44.7)	(42–47.1)
CHIKV infection				
IgG	434 (29.8)	(27.5, 32.3)	218 (29.5)	(26.4, 32.9)
IgM	148 (10.2)	(8.7, 11.8)	35 (4.7)	(3.4, 6.6)
RT-PCR	42 (3.3)	(2.4, 4.4)	-	-
Total Prevalence ^††^	538 (36.8)	(34.3, 39.2)	558 (38.1)	(35.6, 40.6)
DENV infection				
IgG	1255 (86.2)	(84.3, 88)	595 (80.2)	(77.2, 82.9)
IgM	41 (2.8)	(2.1, 4)	82 (11.1)	(9, 13.5)
RT-PCR	51 (4)	(3.1, 5.2)	-	-
Total Prevalence ^††^	1266 (87)	(85.1, 89)	1292 (88.7)	(87, 90.2)

Notes: ^†^ IgM and IgG Antibodies detected by ELISA test and real-time reverse polymerase chain reaction assay (RT-PCR). ^††^ Total prevalence ZIKV infection and CHIKV infection, detected for Antibodies detected by ELISA test and real-time reverse polymerase chain reaction assay (RT-PCR), according to WHO algorithms [22].

**Table 2:** Associated factors with the Prevalence of ZIKV infection in the women of reproductive age cohort, Fortaleza, Brazil, 2018–2019.

Variables	Positive ZIKV ^†^		Crude OR		*p*-Value ^††^
	n (%)	95%CI	OR	95%CI	
**Socioeconomic and demographic**					
Age group (n = 1382)	n = 649				
15 to 19	111 (38.4)	(32.9, 44.1)	1	1	0.005 *
20 to 29	334 (48.9)	(45.2, 52.7)	1.53	(1.16, 2.03) **	
30 to 39	204 (49.8)	(44.9, 54.6)	1.59	(1.17, 2.16) **	
Self-identified race or skin color (n = 1373)	(n = 647)				0.45
White	70 (44.3)	(36.8, 52.1)	1	1	
Non-white	577 (47.5)	(44.7, 50.3)	1.13	(0.81, 1.59)	
Educational Level (n = 1381)					0.01 **
Complete elementary or less	243 (51.9)	(47.4, 56.4)	1	1	
High school or higher	405 (44.4)	(41.2, 47.6)	0.74	(0.59, 0.92)	
Socioeconomic status (n = 1392)	n = 649				
A, B and C1 (Higher)	258 (43.2)	(39.3, 47.2)	1	1	0.05
C2 (Middle)	300 (49.3)	(45.2, 53.2)	1.27	(1.01, 1.60)	
D and E (Lower)	91 (51.7)	(44.3, 59.2)	1.41	(1.02, 2.0)	
Beneficiary of Cash Transfer Program (n = 1382)	n = 649				
Yes	365 (48.4)	(44.8, 51.9)	1	1	0.23
No	284 (45.2)	(41.4, 49.1)	0.88	(0.71, 1.09)	
Beneficiary of State Social Programs (n = 1373)	n = 647				
Yes	377 (48.2)	(44.6, 51.6)	1	1	0.38
No	270 (46.0)	(41.8, 49.8)	0.91	(0.73, 1.13)	
Employment Situation (n = 1382)	n = 649				
Not working or unemployed	411 (45.8)	(42.5, 49)	1	1	0.27
Employed	238 (49.2)	(44.7, 53.6)	1.16	(0.90, 1.48)	
Number of Children (n = 1379)	n = 647				
None	161 (41.0)	(36.1, 46.0)	1	1	0.01 **
1	239 (48.6)	(44.1, 53)	1.36	(1.05, 1.79) **	
2 or more	247 (50.1)	(45.9, 54.6)	1.45	(1.11, 1.90) **	
Household Residents (n = 1381)	n = 649				
<4	257 (44.9)	(40.8, 48.9)	1	1	0.17
≥4	392 (48.5)	(45.2, 52.0)	1.16	(0.93, 1.44)	
**Household characteristics**					
Water source (n = 1375)	n = 646				
Public System	564 (46.1)	(43.3, 48.9)	1	1	0.07
Well or spring	82 (54.0)	(46, 61.7)	1.37	(0.97, 1.92)	
Street Condition (n = 1382)	n = 649				
Paved	546 (48.0)	(44.9, 50.7)	1	1	0.14
Not Paved	103 (43.0)	(36.6, 49.1)	0.81	(0.61, 1.08)	
Water storage needed (n = 1378)	n = 652				
No	438 (45.1)	(42, 48.2)	1	1	0.03 *
Yes	209 (51.5)	(47, 56.3)	1.30	(1.02, 1.63)	
Garbage collection (n = 1368)	n = 642				
Twice a week or more	629 (46.6)	(43.9, 49.3)	1	1	0.05
Once a week	13 (68.4)	(45.1, 85.1)	2.48	(0.78, 0.97)	
**Medical History**					
Pregnant at the interview (n = 1372)	n = 645				
No	546 (49.6)	(46.6, 52.6)	1	1	0.0001 ***
Yes	99 (36.5)	(31.0, 42.4)	0.59	(0.44, 0.77)	
Chikungunya virus infection (n = 1382)	n = 649				
Negative	372 (43.4)	(40.1, 46.7)	1	1	<0.001 ***
Positive	277 (649)	(48.4, 57)	1.45	1.17, 1.81	
Self-report of Family History of Arbovirus Infections ^#^ (n = 1299)	n = 649				
No	218 (39.6)	(35.6, 43.8)	1	1	0.001 ***
Yes	431 (51.8)	(48.4, 55.2)	1.64	(1.31, 2.04)	
**Health-related behaviors**					
Reported prenatal care (1372)	n = 645				
No or not pregnant	553 (49.5)	(46.5, 52.4)	1	1	0.001 ***
Yes	84 (35.3)	(29.5, 41.6)	0.56	(0.42, 0.74)	
Repellent use (1372)	n = 614				
Correctly	87 (38.0)	(32, 44.5)	1	1	0.001 ***
Incorrectly or not use	527 (49.2)	(46.3, 52.2)	1.58	(1.18, 2.12)	
Knowledge about Zika prevention (n = 1382)	n = 649				
None	191 (51.6)	(46.5, 56.7)	1	1	0.03 *
One or more	458 (45.3)	(42.3, 48.3)	0.77	(0.61, 0.98)	

Notes: ^†^ IgM and IgG Antibodies detected by ELISA test and real-time reverse polymerase chain reaction assay (RT-PCR). ^††^ Significant differences detected according to bivariate logistic regression analysis. * *p*v < 0.05; ** *p*v < 0.01; *** *p*v < 0.001. ^#^ Self-report of family history of arbovirus infection Chikungunya virus, dengue virus or Zika virus. OR = odds ratios, CI = confidence intervals.

**Table 3:** Multivariate model of factors associated with the ZIKV infection in the ZIF-cohort of women of reproductive age in Fortaleza, Brazil, 2018–2019.

Variables	Block 1		Block 2		Block 3	
	OR	95%CI	OR	95%CI	OR	95%CI
Age group						
15 to 19	1	1				
20 to 29	1.64	(1.24, 2.19) ***				
30 to 39	1.72	(1.26, 2.35) ***				
Educational Level						
Elementary complete or less	1	1				
Complete High School or higher	0.70	(0.56, 0.90) **				
Water storage						
No	1	1				
Yes	1.29	(1.02, 1.64) *				
Reported prenatal visits						
No or not pregnant			1	1		
Yes			0.60	(0.44, 0.82) **		
Repellent use						
Correctly			1	1		
Incorrectly or not use			1.39	(1.02, 1.88) *		
Family history of arboviruses						
Negative					1	1
Positive					1.42	(1.13, 1.79) *

Notes: Significant differences detected according to multivariate logistic regression analysis; Wald test; *** *p* < 0.001, **; *p* < 0.01 and *; *p* < 0.05. Self-report of family history of arbovirus infection.

## Data Availability

Data are available upon request of corresponding authors for legitimate scientific use.

## Supplementary Materials

The following supporting information can be downloaded at: https://www.mdpi.com/article/10.3390/tropicalmed10030067/s1, Figure S1: Location of the study area and chosen health posts in the neighbourhoods of Barra do Ceará (black and grey dots), Rodolfo Teófilo (green dot) and Conjunto Esperança (blue dot), Fortaleza, Brazil, 2017.
