# Supplementary figures and images for: Syndemic Factors Associated with Zika Virus Infection Prevalence and Risk Factors in a Cohort of Women Living in Endemic Areas for Arboviruses in Northeast Brazil
Source: Trop Med Infect Dis. 2025 Mar 1;10(3):67. doi: 10.3390/tropicalmed10030067 (PMC11945531; doi:10.3390/tropicalmed10030067)

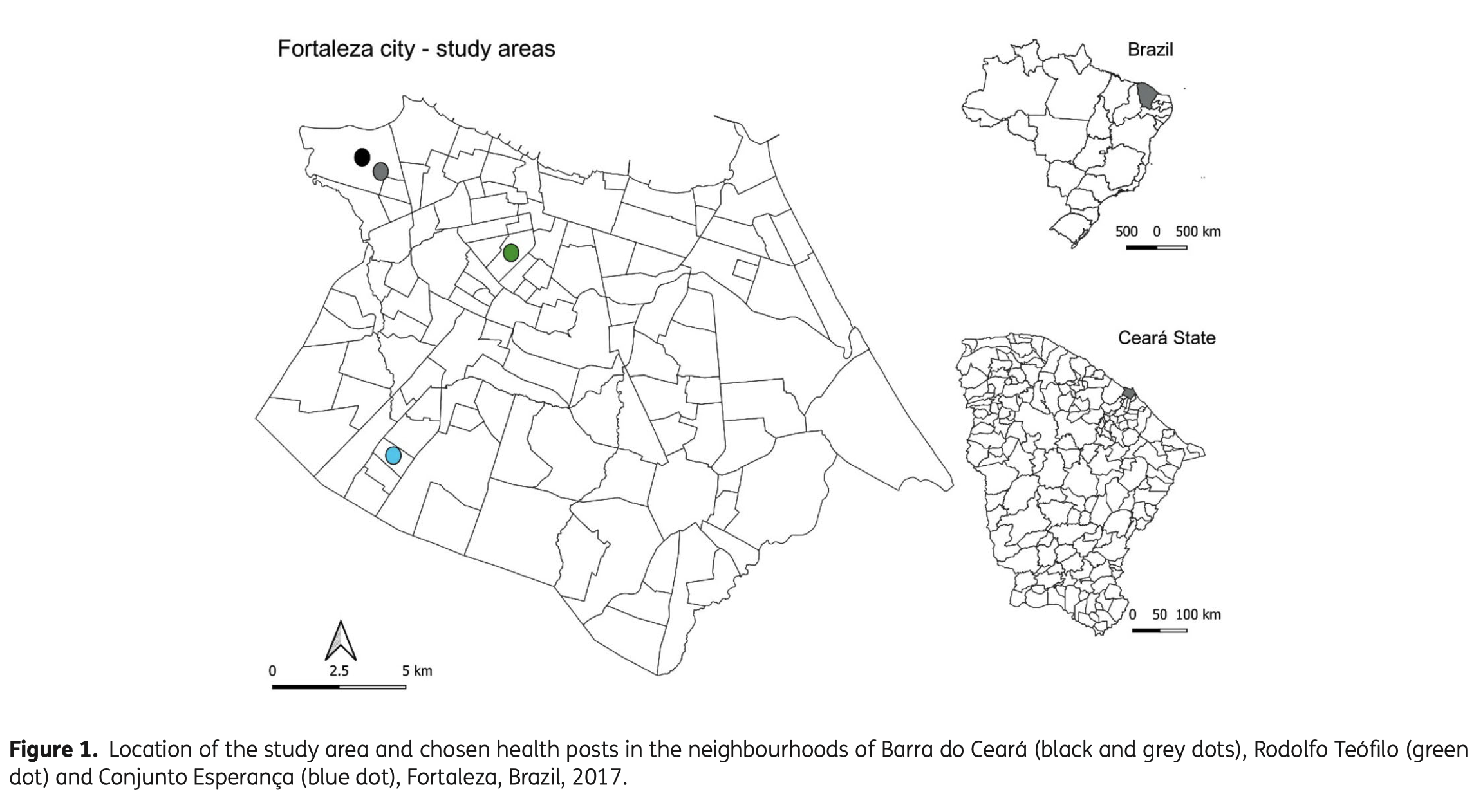

Supplement: Supplementary file 1 [file tropicalmed-10-00067-s001.zip › tropicalmed-3412592-supplementary.png]
